# Global prevalence of *Helicobacter pylori* infection among individuals with obesity: A protocol for a systematic review and meta‐analysis

**DOI:** 10.1002/hsr2.1505

**Published:** 2023-08-21

**Authors:** Alireza Sadeghi, Niloofar Dehdari Ebrahimi

**Affiliations:** ^1^ Student Research Committee Shiraz University of Medical Sciences Shiraz Iran

**Keywords:** bariatric surgery, *Helicobacter pylori*, meta‐analysis, obesity, systematic review

## Abstract

**Background and Aims:**

Modern populations are prone to obesity, as sedentary lifestyles prevail globally. Previous research has shown that obesity and *Helicobacter pylori* are mutually associated. However, the global prevalence of *H. pylori* among individuals with obesity is not yet determined.

**Methods:**

A comprehensive search will be conducted in PubMed, Scopus, and Web of Science online databases for studies that have reported the prevalence of *H. pylori* infection among individuals with obesity. Cross‐sectional, case–control, and cohort studies will be included if reported sufficient data. Records screening, data extraction, and quality assessment will be done by independent reviewers. Joanna Bridge Institute checklist for prevalence studies will be used to appraise the included studies. Prevalence will be pooled using random effect models. Heterogeneity will be quantified by *I* ^2^ and *p* value. Subgroup analyses and meta‐regression will be utilized to address the sources of residual between‐study heterogeneity.

**Discussion:**

Understanding the regional and global occurrence of *H. pylori* infection in individuals with obesity can provide valuable insights for health policymakers and clinicians to devise proficient diagnostic and eradication strategies, thereby enhancing postoperative outcomes for patients undergoing bariatric surgery. The study's strength will lie in not being restricted to language and time of publication, comprehensive investigation of regional and pre‐ and posteradication estimates, and the effects of time trends and sociodemographic indices on *H. pylori* prevalence. However, potential heterogeneity in methodologies used across prevalence studies could affect the interpretation of the results. Additionally, the study relies on previously published studies, limiting data quality and completeness.

## Highlights

- Obesity and *H. pylori* infection are mutually associated, but the global prevalence of *H. pylori* among individuals with obesity is unknown.
- This study aims to conduct a comprehensive search and meta‐analysis of studies reporting the prevalence of *H. pylori* infection among individuals with obesity, regardless of language and time of publication, to provide a more accurate estimate of the global prevalence.
- The results of this study can guide health policymakers and clinicians in developing effective diagnostic and eradication practices for *H. pylori* infection in individuals with obesity, as well as improving postoperative outcomes for bariatric surgery patients.

## 1 INTRODUCTION

World Health Organization (WHO) classifies individuals as obese based on their body mass index (>30). Obesity is a worldwide epidemic and a public health challenge with 13% (11% of men and 15% of women) prevalence globally. In 2016, over 650 million individuals were obese worldwide.^1^ Furthermore, obesity is often accompanied by several comorbidities, causing severe public and personal health‐economic burdens. In this regard, research has extensively investigated the mutual relationship between *Helicobacter pylori* infection and obesity.^2^, ^3^, ^4^, ^5^

The spiral‐shaped, Gram‐negative bacterium known as *H. pylori* colonizes the epithelium of the human stomach. Reports indicate that in 2015, approximately half of the global population (4.4 billion individuals) were infected with *H. pylori*.^6^ The pathogenic impact of *H. pylori* on human health presents in the form of diverse ailments, among which, are peptic mucosa‐associated lymphoma, ulcer diseases, and gastric cancer.^7^, ^8^, ^9^

A recent meta‐analysis has shown that patients infected with *H. pylori* were more likely to be obese and individuals with obesity had higher risks of *H. pylori* infection.^10^ Therefore, individuals with obesity are usually encouraged to eradicate *H. pylori*, especially when undergoing surgeries (among them bariatric surgeries). Several studies have reported that the prevalence of *H. pylori* infection among individuals with obesity ranges between 8.7% and 85.5%.^11^, ^12^ The wide range of the estimates and the absence of a systematic review to comprehensively gather the evidence makes the estimations inconclusive. Therefore, the prevalence of *H. pylori* infection among individuals with obesity is still unknown. Knowing the global and local estimated prevalence of *H. pylori* infection can guide clinicians to acquire sounder therapeutic and diagnostic strategies. Therefore, our study will aim to fill this research gap by conducting a comprehensive systematic review and meta‐analysis of the literature reporting on the prevalence of *H. pylori* infection among individuals with obesity.

## 2 REVIEW QUESTION

The review will be interested in individuals with obesity as the population and those who have concurrent obesity and *H. pylori* as the outcome condition. A comprehensive collection of studies from various regions worldwide will be compiled, specifically seeking favorable data that can be combined to estimate the global prevalence of *H. pylori* among individuals with obesity. Additionally, this study seeks to explore the potential influence of population‐level factors on the relationship between obesity and *H. pylori*. Specifically, aims to determine the prevalence of *H. pylori* in the countries under investigation, categorized according to WHO regional groupings and Sociodemographic Index (SDI) classifications, while also examining how this prevalence varies across different age groups.

## 3 MATERIAL AND METHODS

This protocol for systematic review and meta‐analysis is reported according to the Preferred Reporting Items for Systematic Reviews and Meta‐Analysis protocols (PRISMA‐P) 2015 Guidelines.^13^ A completed PRISMA‐P checklist is provided in the Supporting Information: Material 1.

### 3.1 Data sources and search strategy

The search will be conducted in the PubMed, Scopus, and Web of Science online databases. The search strategy is a combination of “*H. pylori*,” “obesity,” and related keywords and is provided in the Supporting Information: Material 2. The systematic review and meta‐analysis will adhere to the PRISMA guideline, ensuring a rigorous approach to the development, conduct, and reporting of the study.^14^

### 3.2 Screening for eligibility

Two reviewers will independently conduct the primary screening based on the titles and abstracts using Rayyan online tool for managing systematic reviews.^15^ The records that pass the primary screening process will be then assessed for eligibility based on the full articles. Disagreements will be resolved through discussions. In case of unavailable full texts, the reviewers will contact the authors through their email addresses and will await for at least 1 month for responses. In case of missing data, if crucial, the reviewers will contact the authors for clarifications, and if no responses were received, the studies will be excluded from the review.

Published and preprint observational studies (including cohort, cross‐sectional, and case–control studies), posters, and conference abstracts will be eligible for inclusion if reported the prevalence of *H. pylori* among individuals with obesity. No time and language restrictions will be applied to avoid bias.

### 3.3 Data extraction

Data will be extracted into Excel spreadsheets in duplicate. Two reviewers will independently recheck the extracted data to detect any errors. Study‐level characteristics, including first author, publication year, publication type, study year(s), study country, sample size (individuals with obesity), number of individuals with *H. pylori* infection, mean population age, male‐to‐female ratio, criteria for obesity, and detection tests will be extracted from the included studies.

### 3.4 Quality assessment

Joanna Bridge Institute checklist for prevalence studies will be utilized to assess the quality of the included studies by two independent reviewers. Disagreements will be resolved by discussion. The checklist is available in the Supporting Information: Material 3.

### 3.5 Analyses

Stata version 16 (StataCorp) will be utilized for analyses. Using a random‐effects model (DerSimonian‐Laird method), the pooled effect sizes will be reported as percentage and 95% confidence and prediction intervals. Each outcome will be transformed using Freeman–Tukey double arc‐sine method to minimize the bias induced by the effect of extremely high or low data on the pooled estimate.^16^ Cochrane's *Q* statistic, *I* ^2^, and *p*‐value will be used to quantify the residual between‐study heterogeneity. *I* ^2^ of 25%, 50%, and 75% will be interpreted as low, moderate, and high heterogeneity, respectively.^17^ Forest plots will be employed to facilitate visual representations.

Subgroup and meta‐regression analyses will be used to detect the possible sources of residual between‐study heterogeneity and to report the pooled estimates for different conditions of major predictive moderators. Major grouping moderators will be gender ratio, mean age, countries, WHO regional classification, SDI classifications, time the study was conducted, sample size, history of eradication, and diagnostic test. In addition, a subgroup analysis will be done to detect the possible discrepancies between the results of peer‐reviewed versus nonpeer‐reviewed reports. A table will be developed to present the results of subgroup and meta‐regression analyses. Publication bias will be avoided in this systematic review and meta‐analysis, as it has been discouraged for meta‐analyses of proportion studies.^18^

### 3.6 Ethical considerations

Given the absence of human study participants, ethical clearance will not be necessary for conducting this systematic review and meta‐analysis. The findings derived from this review are intended to be communicated through globally recognized scientific journals that adhere to rigorous peer‐reviewed standards.

## 4 DISCUSSION

Previous systematic reviews and meta‐analyses have estimated the worldwide prevalence of obesity and *H. pylori* infection, separately.^1^, ^6^ Nonetheless, the global prevalence of *H. pylori* infection among individuals with obesity remains unknown. Considering a narrative review by Carabotti et al.,^19^ this will be the first systematic review and meta‐analysis on the worldwide prevalence of *H. pylori* infection among individuals with obesity.

An evidence‐supported estimation of the regional and global prevalence of *H. pylori* will be greatly helpful to health policymakers and clinicians as it has been demonstrated that *H. pylori* eradication strategies should be made considering its prevalence among the target population.^20^ Therefore, diagnostic and therapeutic strategies could differ from place to place depending on the prevalence of *H. pylori*. These strategies could include whether any diagnostic tests should be done, the test of choice, and the populations that should be tested.^21^ Consequently, practicing without insufficient evidence can greatly impact the public health economy.

Bariatric surgery is widely recognized as an effective therapeutic intervention for individuals with obesity, particularly those with severe forms of the condition. Before performing this procedure, bariatric surgeons commonly opt to eliminate *H. pylori*, although its necessity remains a subject of debate.^19^ Research has demonstrated that eradicating *H. pylori* before bariatric surgery is associated with improved postoperative outcomes.^19^ Nevertheless, there is ongoing controversy regarding the need for confirmatory post‐eradication testing, resulting in some patients undergoing surgery without complete eradication of *H. pylori*.^22^, ^23^ Consequently, understanding the prevalence of *H. pylori* after eradication, both globally and regionally, could provide valuable insights to surgeons in formulating appropriate strategies.

### 4.1 Strengths and limitations

This study will be the first systematic review and meta‐analysis that measures the global and regional prevalence of *H. pylori* infection among individuals with obesity. The included studies will not be limited by the publishing language, therefore minimalizing the language bias and providing better estimations for regional *H. pylori* prevalence. A meticulous investigation of the data will provide evidence of the effects of regional evaluations, pre‐ and posteradication, time trends, and sociodemographic indices on the prevalence of *H. pylori* among individuals with obesity. However, it is important to acknowledge that prevalence studies often exhibit heterogeneity due to variations in the methodologies employed. Thus, the resulted evidence may be challenging for interpretation. In addition, the study will rely on previously published studies and is therefore limited by the quality and completeness of the data reported in those studies.

## 5 REPORTING OF THIS REVIEW

The proposed systematic review will be reported following the PRISMA guidelines. We intend to publish a PRISMA checklist alongside the final report.

## 6 POTENTIAL AMENDMENTS

Amendments are not intended. However, inevitable amendments will be documented and reported transparently.

## AUTHOR CONTRIBUTIONS

**Alireza Sadeghi**: Conceptualization; investigation; methodology; resources; supervision; writing—review and editing. **Niloofar Dehdari Ebrahimi**: Data curation; investigation; resources; writing—original draft; writing—review and editing.

## CONFLICT OF INTEREST STATEMENT

The authors declare no conflict of interest.

## TRANSPARENCY STATEMENT

The lead author Alireza Sadeghi affirms that this manuscript is an honest, accurate, and transparent account of the study being reported; that no important aspects of the study have been omitted; and that any discrepancies from the study as planned (and, if relevant, registered) have been explained.

## Supporting information

Supporting Information 1: PRISMA‐P Flow Diagram.

Supporting Information 2: Search Strategy.

Supporting Information 3: JBI Checklist for Critical Appraisal of Prevalence Studies.

## Data Availability

Data sharing not applicable to this article, as no data sets were generated or analyzed during the current study.
