# Supplementary material for: Global prevalence of Helicobacter pylori infection among individuals with obesity: A protocol for a systematic review and meta‐analysis
Source: Health Sci Rep. 2023 Aug 21;6(8):e1505. doi: 10.1002/hsr2.1505 (PMC10442524; doi:10.1002/hsr2.1505)
Supplement: Supplementary file 2 — Supporting Information 2: Search Strategy. [file HSR2-6-e1505-s002.docx]

S2: Search strategy

# PubMed

(((Helicobacter[Title/Abstract] AND pylori[Title/Abstract]) OR "H. Pylori"[Title/Abstract] OR "H pylori"[Title/Abstract] OR Hpylori[Title/Abstract]) AND (obesity[Title/Abstract] OR obese[Title/Abstract] OR bariatric[Title/Abstract])) OR ("Helicobacter pylori"[Mesh] AND ("Obesity"[Mesh] OR "Bariatric Surgery"[Mesh]))

**Results: 718**

**Date: 2023-05-19**

# Scopus

TITLE-ABS-KEY((Helicobacter AND pylori) OR "H. Pylori" OR "H pylori" OR Hpylori) AND TITLE-ABS-KEY(obesity OR obese OR bariatric)

**Results: 1,375**

**Date: 2023-05-19**

# Web of Science

TS=((Helicobacter AND pylori) OR "H. Pylori" OR "H pylori" OR Hpylori) AND TS=(obesity OR obese OR bariatric)

**Results: 1,088**

**Date: 2023-05-19**

**Duplicates: 1,015**
